# Molecular Characterization, Tumor Microenvironment Association, and Drug Susceptibility of DNA Methylation-Driven Genes in Renal Cell Carcinoma

**DOI:** 10.3389/fcell.2022.837919

**Published:** 2022-03-21

**Authors:** Jinpeng Wang, Wei Zhang, Wenbin Hou, Enyang Zhao, Xuedong Li

**Affiliations:** Department of Urology, The Second Affiliated Hospital of Harbin Medical University, Harbin, China

**Keywords:** renal cell carcinoma, molecular characterization, methylation-driven genes, tumor microenvironment, drug susceptibility

## Abstract

Accumulating evidence suggests that DNA methylation has essential roles in the development of renal cell carcinoma (RCC). Aberrant DNA methylation acts as a vital role in RCC progression through regulating the gene expression, yet little is known about the role of methylation and its association with prognosis in RCC. The purpose of this study is to explore the DNA methylation-driven genes for establishing prognostic-related molecular clusters and providing a basis for survival prediction. In this study, 5,198 differentially expressed genes (DEGs) and 270 DNA methylation-driven genes were selected to obtain 146 differentially expressed DNA methylation-driven genes (DEMDGs). Two clusters were distinguished by consensus clustering using 146 DEMDGs. We further evaluated the immune status of two clusters and selected 106 DEGs in cluster 1. Cluster-based immune status analysis and functional enrichment analysis of 106 DEGs provide new insights for the development of RCC. To predict the prognosis of patients with RCC, a prognostic model based on eight DEMDGs was constructed. The patients were divided into high-risk groups and low-risk groups based on their risk scores. The predictive nomogram and the web-based survival rate calculator (http://127.0.0.1:3496) were built to validate the predictive accuracy of the prognostic model. Gene set enrichment analysis was performed to annotate the signaling pathways in which the genes are enriched. The correlation of the risk score with clinical features, immune status, and drug susceptibility was also evaluated. These results suggested that the prognostic model might be a promising prognostic tool for RCC and might facilitate the management of patients with RCC.

## Introduction

Renal cell carcinoma (RCC) is the most common urologic cancer type. There were an estimated nearly 65,340 new cases and 14,970 deaths worldwide in 2020 (Hsieh et al., 2018). With the advancement of diagnostic approaches, an increasing number of RCC could be diagnosed at early-stage (Alonso-Gordoa et al., 2019). Early diagnosis of RCC is essential for prolonging the overall survival (OS) of patients (Porta et al., 2016). Currently, the most curative treatment for localized RCC is still considered to be surgical resection (Ljungberg et al., 2010). Nowadays, there are already many surgical methods to remove tumors, including nephron-sparing surgery, radical nephrectomy, and laparoscopic surgery (Ljungberg et al., 2010). However, the treatment options are still limited for unresectable and metastatic RCC (Li et al., 2016; Shinder et al., 2017). Early and accurate diagnosis of RCC has been regarded as a research priority (Porta et al., 2016). Recently, with biomedical research progresses, molecular prognostic biomarkers have become one of the basic ideas of precision medicine. Unfortunately, early-stage diagnosis for RCC by molecular prognostic biomarkers has many challenges due to the lack of biomarkers for the prediction of progression (Li et al., 2018). For this reason, more molecular biomarkers are urgently needed to screen for RCC diagnosis.

Modifications of the epigenome, such as DNA methylation, play a crucial role in the development of many diseases (Shen et al., 2010). The correct methylation pattern is very important for normal biological functions, and aberrant methylation is one of the drivers for the progression of several diseases, especially cancer (Jeltsch and Jurkowska, 2016). Numerous prior studies suggested that hypermethylated and hypomethylated DNA always show different activities (Park et al., 2011). Abnormal methylation always occurs in cancer cells, leading to some genes being aberrantly activated and some genes being aberrantly silenced (Vasanthakumar et al., 2013). Hypomethylation of proto-oncogenes or tumor suppressor gene methylation is considered one of the leading mechanisms of tumorigenesis in many cancer types (Hayslip and Montero, 2006; Ding et al., 2020). Therefore, the detection of the methylation pattern alteration of specific genes can aid the cancer diagnosis. Silencing of tumor suppressor genes caused by promoter hypermethylation provides new ideas for inquiring about the molecular mechanisms of RCC (Torres-Ferreira et al., 2017). The aberrant methylation is involved in the progression of RCC. Some studies found that DNA methylation in RCC silenced the von Hippel–Lindau (VHL) tumor suppressor gene (Grech et al., 2015). In addition, RCC can be genotyped based on DNA methylation mutations (Tian et al., 2014).

There have been many studies focused on DNA methylation or gene expression. However, RCC prognostic models based on DNA methylation-driven genes have barely been explored. In this study, we established a prognostic model to accurately predict patient survival. In addition, we divided RCC samples into two clusters according to 146 DEMDGs and further explored the relationship between the tumor immune status and clusters of RCC. The results we distilled will ultimately contribute to improving the diagnostic accuracy and efficacy in immunotherapy.

## Materials and Methods

### Date Collection

A total of 28 RNA-seq transcriptional profiling of normal samples were downloaded from the GTEx (Genotype-Tissue Expression) dataset (https://gtexportal.org/). A total of 1021 RNA-seq transcriptome profiling (128 normal samples and 893 RCC samples), 872 DNA methylation data (205 normal samples and 667 tumor samples), and corresponding clinical information of RCC were downloaded from TCGA (The Cancer Genome Atlas) dataset (https://gdc.cancer.gov/).

### Differentially Expressed Genes Screening in RCC and Heatmap Plotting

We standardized RNA-seq transcriptional profiling by using the “limma” R package, and the Wilcoxon rank-sum test was utilized to identify DEGs (Ritchie et al., 2015; Zhang et al., 2020). The false discovery rate (FDR) < 0.05 and |log_2_ fold change (FC)| > 2 were taken advantage of as cutoff criteria. The “pheatmap” R package was used to plot the heatmaps (Li et al., 2020).

### Integrated Analysis of Gene Methylation Data and Gene Expression Data

Gene expression data and DNA methylation data were standardized by using the “limma” R package (Ritchie et al., 2015). DNA methylation-driven genes (MDGs) were identified using the “methylMix” R package (Gevaert, 2015; Zhang et al., 2020). DNA methylation data and paired gene expression data were integrated and analyzed jointly to identify the DNA methylation status negatively correlated with the gene expression of a particular gene, indicating that the gene is a DNA methylation-driven gene (Cedoz et al., 2018). Inclusion criteria were set to the correlation of methylation data and corresponding gene expression data of DEGs less than −0.3, |log_2_FC| > 0 and adjust *p* < 0.05. The differentially expressed DNA methylation-driven genes (DEMDGs) were obtained by intersecting MDGs and DEGs for further analysis (Zhang et al., 2020).

### Construction of the PPI Network

The PPI network was established by the Search Tool for the Retrieval of Interacting Genes/Proteins (STRING) database (https://string-db.org/) and visualized by Cytoscape software (v3.8.2) (Shannon et al., 2003). To generate an interaction network, 146 DEMDGs were uploaded to the STRING database. The obtained networks were downloaded in a tabular format and uploaded to Cytoscape for network visualization.

### Evaluation of the Immune Status and Boxplot Plotting

The immune cell infiltration levels in the samples were evaluated by the “CIBERSORT” R package, and the inclusion criteria were *p* < 0.05 (Newman et al., 2015). The stromal score and immune score were also estimated by the “estimate” R package (Jia et al., 2018). Boxplots were plotted using the “ggpubr” R package (Whitehead et al., 2019).

### Consensus Clustering Analysis

We capitalized on the k-means clustering algorithm in the “ConsensusClusterPlus” R packet to perform clustering (Wilkerson and Hayes, 2010). Here, we performed the partition around medoids clustering algorithm and Euclidean distance. The cluster number was tested from 2 to 9, and the optimal one was selected to produce the most stable consensus matrix and the least overlapping cluster assignments across permuted clustering runs. We implemented it with the R package ConsensusClusterPlus.

### Construction of the Prognostic Model and Validation

Utilizing the “glmnet” R package, “survival” R package, and “survminer” R package, the prognostic model was constructed (Armbruster et al., 2019; Wang et al., 2021; Xi et al., 2021). The risk scores were calculated according to a linear combination of the gene expression levels weighted by the regression coefficients from the multivariate Cox regression analysis (Wei et al., 2020). The “survivalROC” R package was utilized to validate the stability of the prognostic model (Huang et al., 2017). The Kaplan–Meier survival curves were carried out to assess the survival time between high- and low-risk score RCC patients (Lawrie et al., 1982). We validated the accuracy of the optimal cut-off value by the principal component analysis (Kim et al., 2021). The Human Protein Atlas database (https://www.proteinatlas.org/) was explored to investigate the expression level of the prognostic genes.

### Total RNA Extraction and Real-Time Quantitative PCR

To evaluate the expression level of mRNA, total RNA was extracted from the cell using TRIzol (Invitrogen). One microgram of total RNA was used as a template for cDNA synthesis using a PrimeScript RT Reagent Kit (Takara). Real-time quantitative PCR (qRT-PCR) was performed in a reaction mixture containing SYBR Green (Takara) with the CFX96 Touch real-time PCR detection system (Bio-Rad). The expression level of related mRNAs was calculated using 2^−ΔΔCT^, and the related GAPDH mRNA expression was used as an endogenous control. The primer sequences involved in this study are shown in Supplementary Table S1. Each PCR reaction was performed in triplicates.

### Independence of the Prognostic Model From Clinical Features

We used the “survival” R package to evaluate the independence of the prognostic model from clinical features *via* univariate and multivariate Cox regression analyses (Qi et al., 2021). The significant levels were set to *p* < 0.05, and hazard ratios (HRs) with 95% CIs were also calculated.

### Construction of the Nomogram and the Dynamic Nomogram

The nomogram was constructed utilizing the “rms” R package (Pond et al., 2014). The web-based survival rate calculator was established using the “shiny” and “DynNom” R packages to predict cancer-specific survival rates dynamically (Sun et al., 2020; Bakin et al., 2021). Calibration curves, which plot the average Kaplan–Meier evaluation according to the corresponding nomogram for 1-, 3-, or 5-year predicted overall survival, are provided to estimate the accuracy of the nomogram.

### Gene Set Enrichment Analysis and Column Diagram Plotting

Gene set enrichment analysis (GSEA) was performed using GSEA4.0 (https://www.gsea-msigdb.org/gsea/index.jsp/). The annotated gene set files (c2.cp.kegg.v7.4. symbols.gmt gene set and c5.go.bp.v7.4. symbols.gmt) were considered as the reference gene set. The inclusion criteria were *p* < 0.05 and FDR < 0.25. The column diagrams were plotted by GraphPad Prism 7 (Elzein et al., 2021).

### Functional and Pathway Enrichment Analysis

Gene Ontology (GO) and Kyoto Encyclopedia of Genes and Genomes (KEGG) analysis were performed using the “enrichplot” R package, “org.Hs.eg.db” R package, and “clusterProfiler” R package (Yu et al., 2012; Zhang et al., 2019). The inclusion criteria were set to *p* < 0.05 and q < 1. The results were visualized by the “ggplot2” R package (Sun et al., 2011).

### Statistical Analysis

All statistical tests were performed by R statistical software (version 4.0.3) (http://www.r-project.org/) using Mann-Whitney testing for continuous data and Fisher’s exact testing for categorical data. The correlation between two continuous variables was measured by Pearson’s correlation coefficient. The hazard ratio (HR) and 95% confidence intervals (CI) were estimated by a Cox regression model using the survival package. Survival analysis was carried out using Kaplan–Meier methods. Differential methylation was calculated from mean (β-value-cancer)—mean (β-value- normal). The differences in variables among different groups were compared by means of the Student’s t-test. *p* < 0.05 was considered to be statistically significant.

## Results

### Identification of 146 DEMDGs in RCC

**FIGURE 1:**
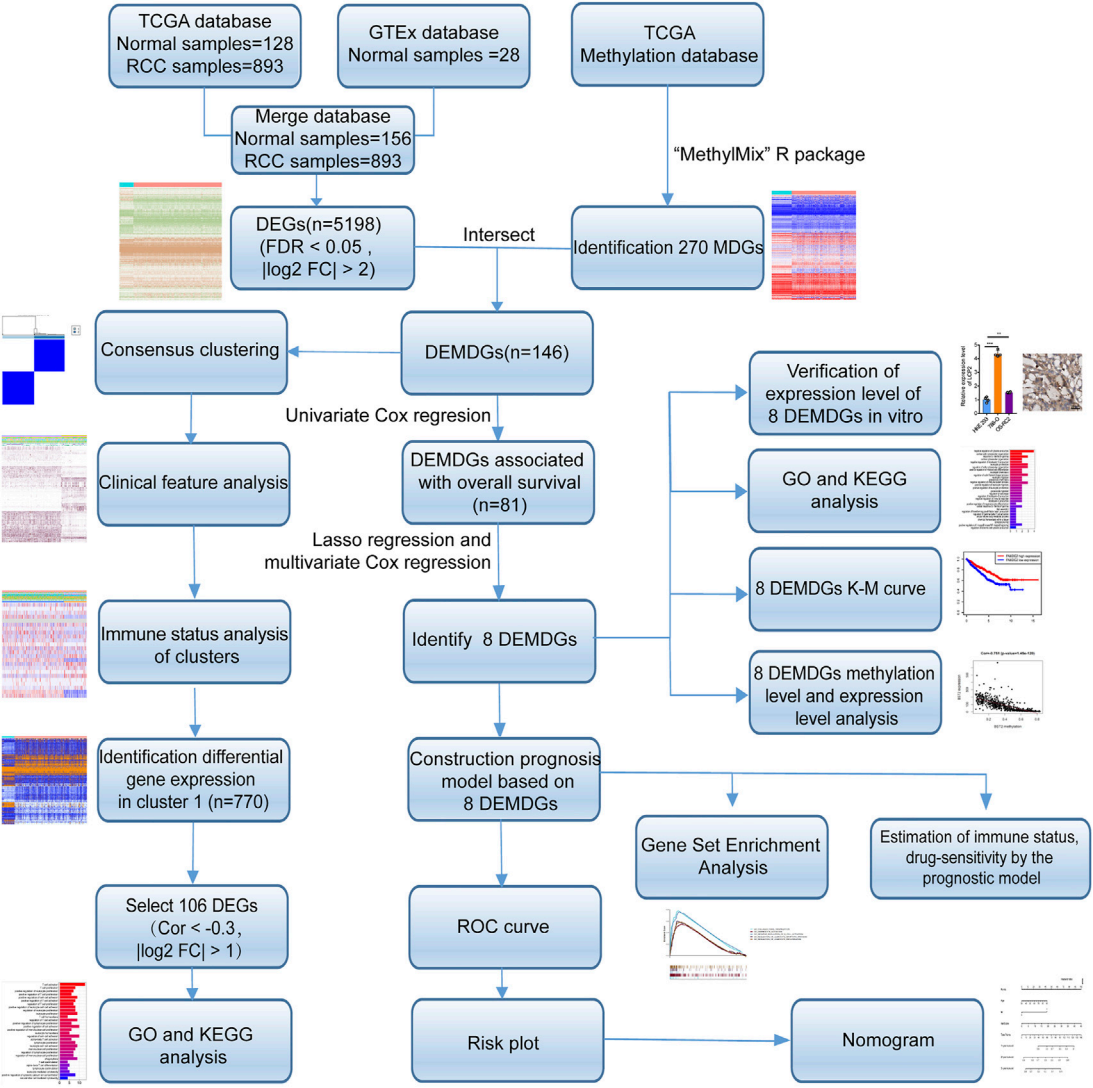
Flow diagram. TCGA, The Cancer Genome Atlas; GTEx, genotype-tissue expression; DEGs, differentially expressed genes; MDGs, methylation-driven genes; DEMDGs, differentially expressed methylation-driven genes; KEGG, Kyoto Encyclopedia of Genes and Genomes; GO, gene ontology; K–M, Kaplan–Meier.

**FIGURE 2:**
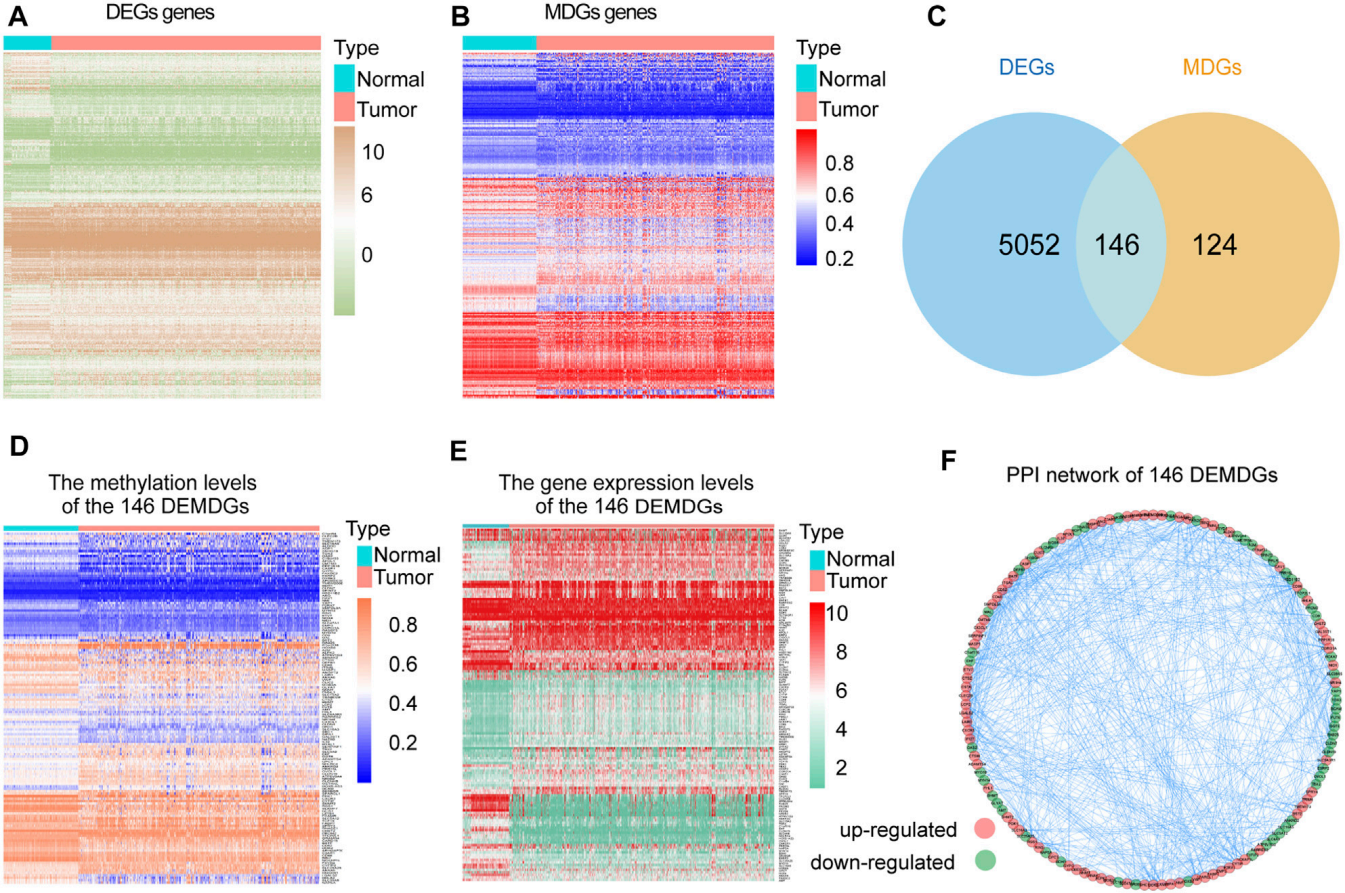
Screening of 146 DEMDGs. **(A)** Heatmap of DEGs in normal samples and RCC samples. **(B)** Heatmap of MDGs in normal samples and RCC samples. **(C)** Venn diagram for 146 DEMDGs in normal samples and RCC samples. The blue circle represents 5,198 DEGs, and the yellow circle represents 270 MDGs. **(D)** Heatmap of methylation levels of 146 DEMDGs. **(E)** Heatmap of gene expression levels of 146 DEMDGs. **(F)** PPI network of 146 DEMDGs.

The research process of the study was shown in Figure 1. Based on the Wilcoxon rank test, a total of 5,198 DEGs were selected from 28 RNA-seq transcriptome profiling of normal samples in the GTEx dataset and 1021 RNA-seq transcriptome profiling (893 RCC samples and 128 normal samples) in TCGA dataset (FDR <0.05 and |log_2_FC| > 2). The heatmap shows the expression of DEGs between RCC samples and normal samples (Figure 2A). We screened the 270 methylation-driven genes (MDGs), whose methylation status negatively correlated with expression levels (Cor < −0.3, |log_2_FC| > 0 and adjust *p* < 0.05) (Supplementary Table S2). The heatmap shows the expression of MDGs between RCC samples and normal samples (Figure 2B). Then, 270 MDGs and 5,198 DEGs were intersected to obtain 146 DEMDGs for further analysis (Figure 2C). We further visualized the methylation levels (Figure 2D) and gene expression levels (Figure 2E) of 146 DEMDGs in RCC samples and normal samples. The comprehensive landscape of DEMDG interactions and DEMDG connection for RCC patients was depicted with the DEMDG network (Figure 2F). The aforementioned results found 146 DEMDGs in RCC and normal samples. These abnormal DEMDGs were interconnected and may be involved in the occurrence and development of RCC.

### Consensus Clustering Based on 146 DEMDGs and Immune Status Analysis of Clusters

**FIGURE 3:**
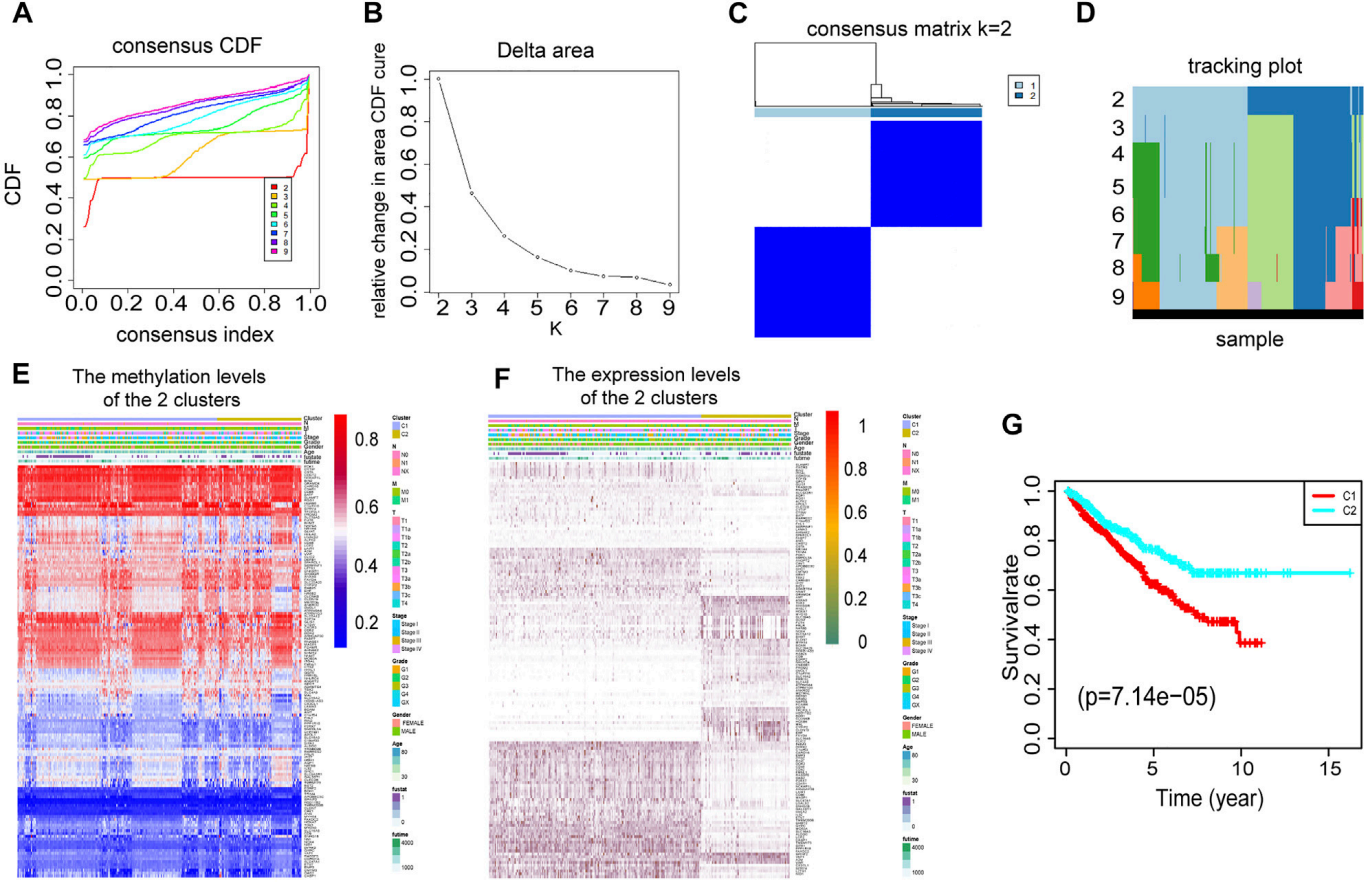
Consensus clustering based on 146 DEMDGs. **(A)** CDF for RCC. **(B)** The area under the CDF curve in RCC. **(C)** Consensus clustering matrix for RCC at *k* = 2. **(D)** Tracking plot for k from 2 to 9. **(E)** Heatmap of methylation levels of 146 DEMDGs in the two clusters, and the distribution of clinical features was compared in the two clusters. **(F)** Heatmap of gene expression levels of 146 DEMDGs in the two clusters, and the distribution of clinical features was compared in the two clusters. **(G)** The survival curves showed significant prognostic differences in the two clusters.

To select the optimized cluster number, we calculated the k-means clustering algorithm with the ConsensusClusterPlus R packet. K = 2 was identified with the optimal clustering stability (Figures 3A–D). Then, we analyzed the methylation levels and gene expression levels of 146 DEMDGs, as well as the clinical features of paired patients. There were significant differences in the methylation levels and gene expression levels between cluster 1 and cluster 2, and the clinical features were evenly distributed in two clusters (Figures 3E,F). The RCC patients in cluster 2 (*n* = 419) had better overall survival (OS) than the patients in cluster 1 (*n* = 435, *p* < 0.001) (Figure 3G).

**FIGURE 4:**
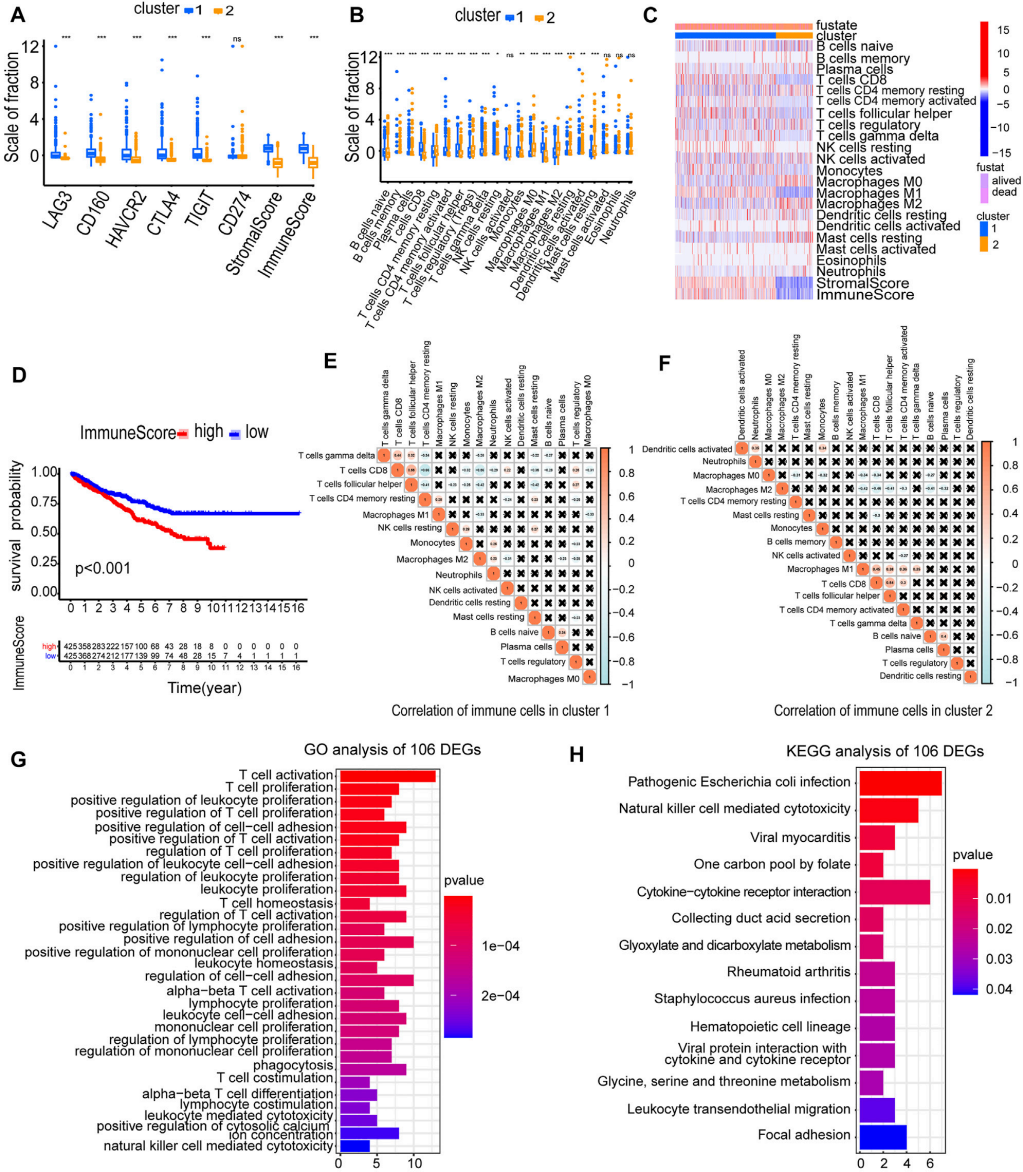
Immune status analysis of two clusters and functional enrichment analysis of 106 DEGs. **(A)** Expression of immune checkpoints in two clusters of RCC. **(B)** The abundance of immune cells in two clusters of RCC. **(C)** The heatmap of the abundance of immune cells in two clusters. **(D)** The survival curves of high and low immune score groups showed significant prognostic differences. **(E)** Correlation matrix of infiltrating immune cells in cluster 1. **(F)** Correlation matrix of infiltrating immune cells in cluster 2. The numbers in the two matrices represent the Pearson correlation coefficient. The coral circle represents a positive correlation, the blue circle represents a negative correlation, and the white circle represents no correlation between two kinds of cells. **(G)** GO term enrichment in the biological process of 106 DEGs. **(H)** KEGG enrichment analysis of 106 DEGs. *p < 0.05; **p < 0.01; and ***p < 0.001.

Immune checkpoint inhibitors (ICIs) are administered for the treatment of RCC. We investigated whether the two clusters were related to ICI-related biomarkers. The results showed that cluster 1 was positively correlated with the high expression of LAG3 (*p* < 0.001), CD160 (*p* < 0.001), HAVCR2 (*p* < 0.001), CTLA4 (*p* < 0.001), and TIGIT (*p* < 0.001), and the stromal score and immune score were significantly higher in cluster 1 than in cluster 2 (*p* < 0.001) (Figure 4A). The abundance of naive B cells (*p* < 0.001), CD8 T cells (*p* < 0.001), CD4 memory-activated T cells (*p* < 0.001), follicular helper T cells (*p* < 0.001), gamma delta T cells (*p* < 0.001), and macrophages M1 (*p* < 0.001) was significantly higher in cluster 1 than in cluster 2 (Figure 4B). The higher immune infiltration level corresponded to cluster 1, and the lower immune infiltration level corresponded to cluster 2 (Figure 4C). The RCC patients in high-immune score groups had a worse OS than the patients in low-immune score groups (*p* < 0.001) (Figure 4D). Furthermore, we accessed the correlation of immune cells in cluster 1 and cluster 2. In cluster 1, the positive correlation between CD8 T cells and follicular helper T cells was the strongest, in which the correlation coefficient was 0.55. The correlation coefficient between CD8 T cells and CD4 memory-resting T cells was −0.66, which was the lowest negative correlation (Figure 4E). However, in cluster 2, the cells with the strongest negative correlation were activated CD8 T cells and macrophages M2, in which the correlation coefficient was −0.46 (Figure 4F). These results showed that the two clusters based on 146 DEMDGs were closely associated with prognosis and immune status in RCC patients.

To explore the possible reasons causing worse OS in cluster 1, we selected 106 DEGs from two clusters (Cor < −0.3 and |log_2_FC| > 1). We used a heatmap visualizing the gene expression levels of 106 DEGs in RCC and normal samples (Supplementary Figure S1). We performed GO and KEGG analysis to analyze underlying functions and pathways of 106 DEGs (*p* < 0.05). Results of GO analysis were significantly enriched in regulation of T-cell activation, T-cell proliferation, positive regulation of leukocyte proliferation, positive regulation of T-cell proliferation, positive regulation of cell–cell adhesion, etc. (Figure 4G; Supplementary Figures S2A, S2B). Results of the KEGG pathways were significantly enriched in pathogenic *Escherichia coli* infection, natural killer cell-mediated cytotoxicity, viral myocarditis, one carbon pool by folate, cytokine–cytokine receptor interaction, etc. (Figure 4H). The aforementioned results indicated that the 106 DEGs were in close contact with the immune microenvironment, which may be the cause for the OS difference between the two clusters.

### Construction and Evaluation of the Prognostic Model

**FIGURE 5:**
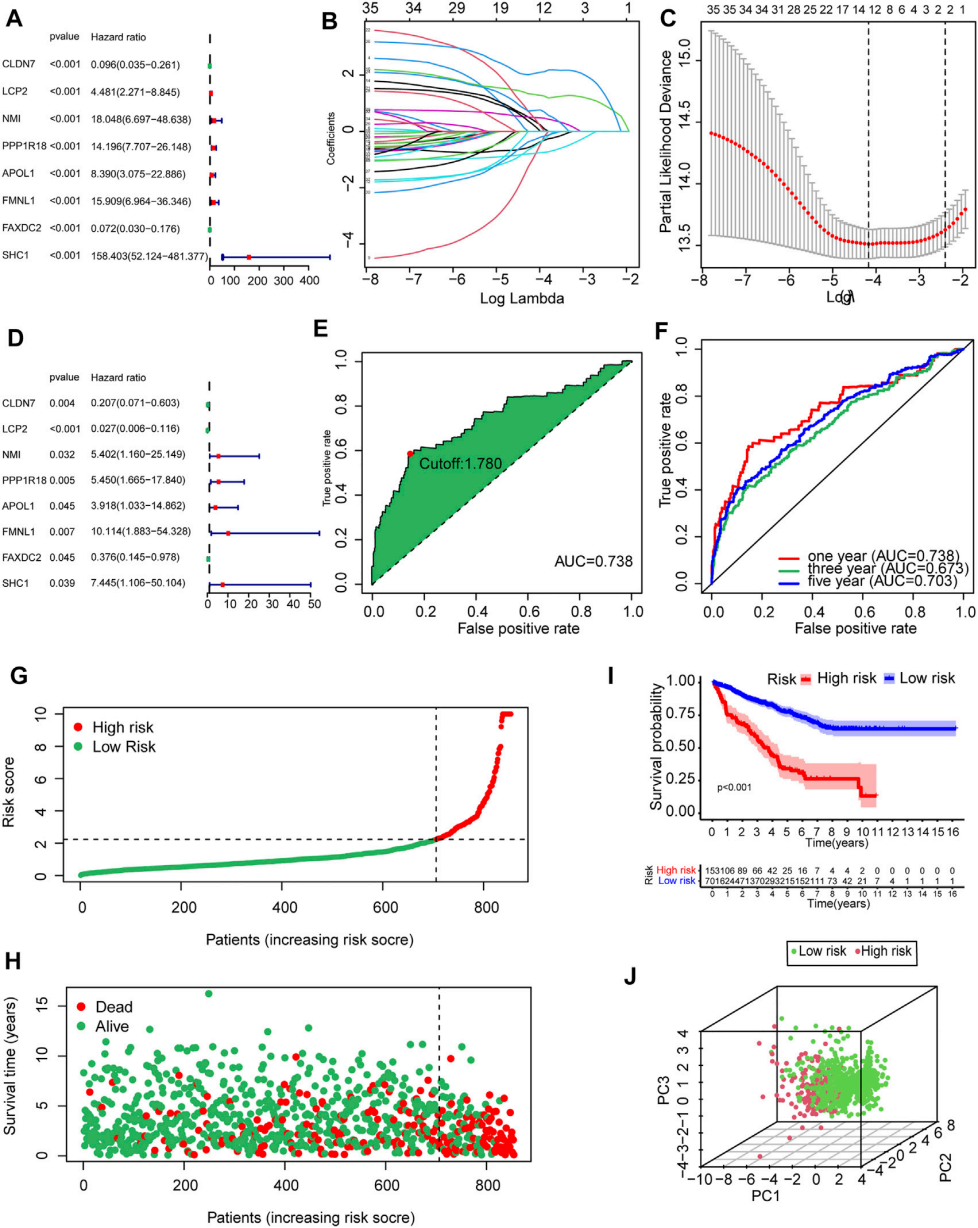
Establishment and validation of the prognostic model based on 146 DEMDGs. **(A)** The hazard ratios (HR) and 95% confidence intervals (CI) of eight DEMDGs in RCC were computed by univariate Cox regression analysis. **(B)** The changing trajectory of each independent variable. **(C)** Confidence intervals for each optimal lambda. 10-fold cross-validation for the tuning parameter selection in the LASSO model. **(D)** The HR and 95% CI of eight DEMDGs in RCC were computed by multivariate Cox regression analysis. **(E)** Risk score for 893 RCC samples; the maximum inflection point is the cut-off point accessed by the AIC. **(F)** The ROC curve for 1-, 3-, and 5-year overall survival. **(G, H)** Distribution of the risk scores of RCC patients. **(I)** The survival curves were plotted to show the survival difference based on the risk score. **(J)** Principal component analysis was performed on RCC samples based on risk scores.

To determine the prognostic value of 146 DEMDGs, univariate Cox regression analysis, LASSO, and multivariate Cox regression analysis were used to identify them. Subsequently, the prognostic model was constructed based on eight independent and prognostic DEMDGs (including LCP2, PPP1R18, APOL1, FMNL1, CLDN7, NMI, FAXDC2, and SHC1) (Figures 5A–D). We analyzed the association between the gene expression and the survival of the patients. The patients’ OS with high expressions of LCP2, PPP1R18, APOL1, FMNL1, NMI, and SHC1 were worse than the low-expression groups (*p* < 0.05), while the patients’ OS with high expressions of CLDN7 and FAXDC2 were better than the low-expression groups (*p* < 0.05) (Supplementary Figure S3). The methylation levels of eight DEMDGs were inversely correlated with their expression levels (*p* < 0.001) (Supplementary Figure S4). The risk score was calculated as follows = LCP2 * (−3.619) + CLDN7 * (−1.577) + FAXDC2*(-0.977) + APOL1*(1.365) + NMI *(1.686) + PPP1R18 * (1.695) + SHC1 * (2.007) + FMNL1 * (2.313). The coefficients of each gene are shown in Table 1.

**TABLE 1:** Multivariate Cox regression analysis of eight DEMDGs.

Id	Coef	HR	HR.95L	HR.95H	*p* Value
CLDN7	−1.577	0.206	0.07	0.602	0.003
LCP2	−3.619	0.026	0.006	0.115	0.00E + 00
NMI	1.686	5.401	1.16	25.149	0.031
PPP1R18	1.695	5.449	1.664	17.839	0.005
APOL1	1.365	3.917	1.032	14.862	0.044
FMNL1	2.313	10.114	1.882	54.327	0.006
FAXDC2	−0.977	0.376	0.144	0.977	0.044
SHC1	2.007	7.444	1.106	50.104	0.039

Coef, coefficient; HR, hazard ratio; CI, confidence interval.

RCC patients were split into high- and low-risk groups according to the optimal cut-off value of the risk score (cutoff = 1.78) (Figure 5E). The AUC of the ROC curves was 0.738, 0.673, and 0.703 within 1, 3, and 5 years, which demonstrated that the risk score had a good prognostic value (Figure 5F). The distributions of the risk score in high- and low-risk groups were shown in Figure 5G. Patients’ mortality risk increased with increasing risk scores (Figure 5H). The survival curve was carried out to assess the survival time between high- and low-risk score groups. The survival time of high-risk groups was significantly worse than the low-risk groups (*p* < 0.001) (Figure 5I). RCC samples were clearly structured in two different groups by the principal component analysis, which suggested our study could significantly reflect the prognosis differences of RCC patients (Figure 5J).

### Verification of the Expression Level of Eight DEMDGs *In Vitro*

**FIGURE 6:**
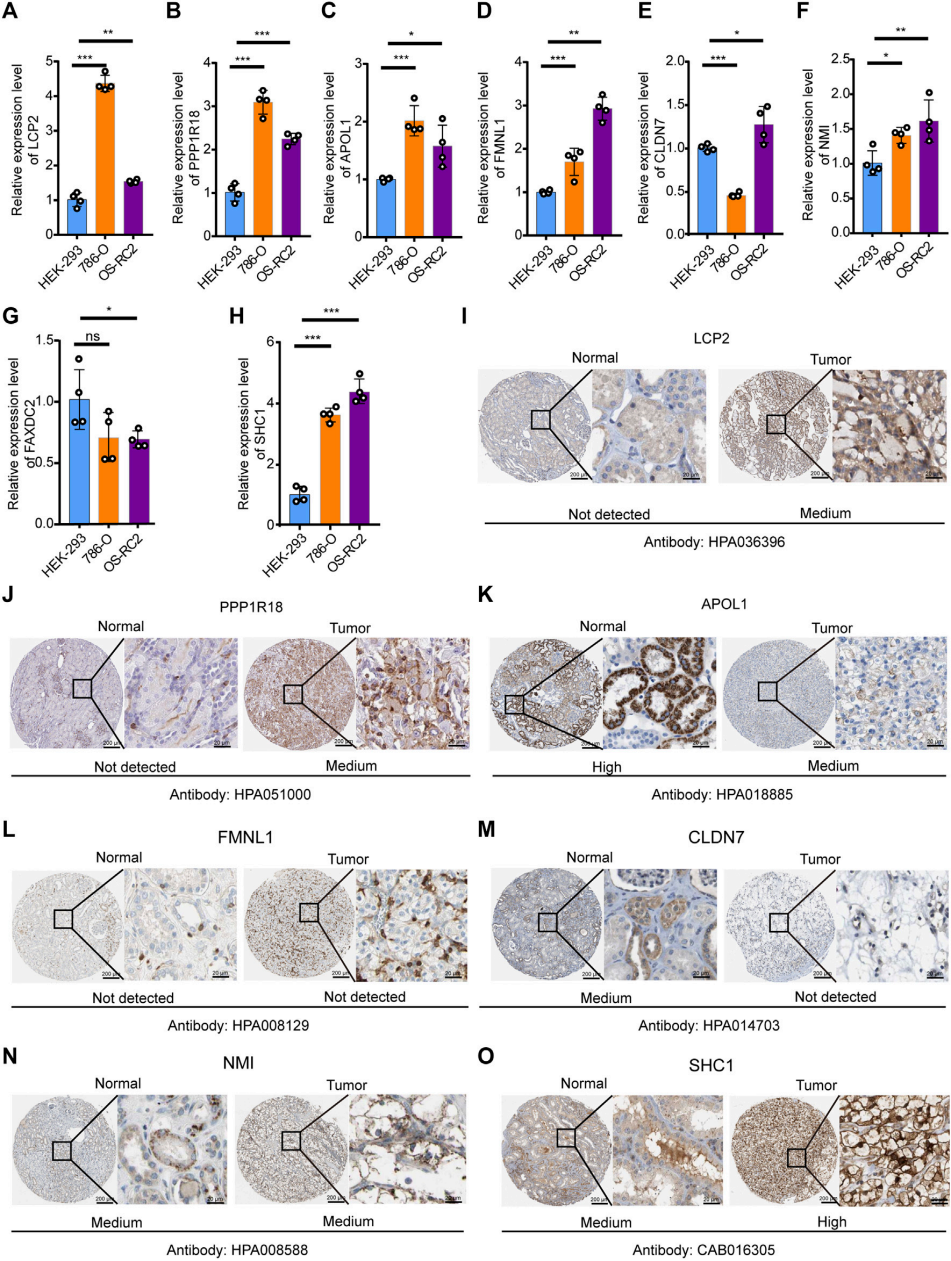
Verification of expression levels of eight DEMDGs *in vitro*. Verification of expression levels of eight DEMDGs *in vitro* **(A–H)** Expression levels of eight DEMDGs in HEK-293 cells and RCC cells. **(I–O)** The representative protein expression of the eight DEMDGs in normal kidney tissue and RCC. Data were from the Human Protein Atlas database. FAXDC2 was not found in the database. *p < 0.05, **p < 0.01, ***p < 0.001. ns, no sense.

To verify the expression levels of eight DEMDGs in RCC cells, we used RT-qPCR analysis to detect human embryonic kidney-293 (HEK-293) and human RCC cell lines (786-O and OS-RC2 cells) (Figures 6A–H). Among them, six DEMDGs (LCP2, PPP1R18, APOL1, FMNL1, NMI, and SHC1) were upregulated in both RCC cells, combined with Supplementary Figure S3; their high expression was associated with poor survival, considering that they might play a role as proto-oncogenes. FAXDC2 was downregulated in both RCC cells. However, CLDN7 was downregulated in 786-O cells and upregulated in OS-RC2 cells. To determine the clinical relevance of these eight gene expressions, HPA clinical specimens were used to analyze the proteins’ expression encoded by these eight genes (Figures 6I–O). Relative to its expression level in normal kidney tissue, SHC1 were strongly positive, while LCP2, PPP1R18, and NMI were moderately positive in RCC tissues. FMNL1 and CLDN7 were not detected in RCC tissues. APOL1 was expressed more abundantly in the normal tissue than in malignant. FAXDC2 was not found on the website. However, our results on APOL1 are contrary to the database. Our team speculated that the main reason was that the data from TCGA database came from all pathological types of RCC, hence the inconsistent results.

### Enrichment Analysis of the Prognostic Model

**FIGURE 7:**
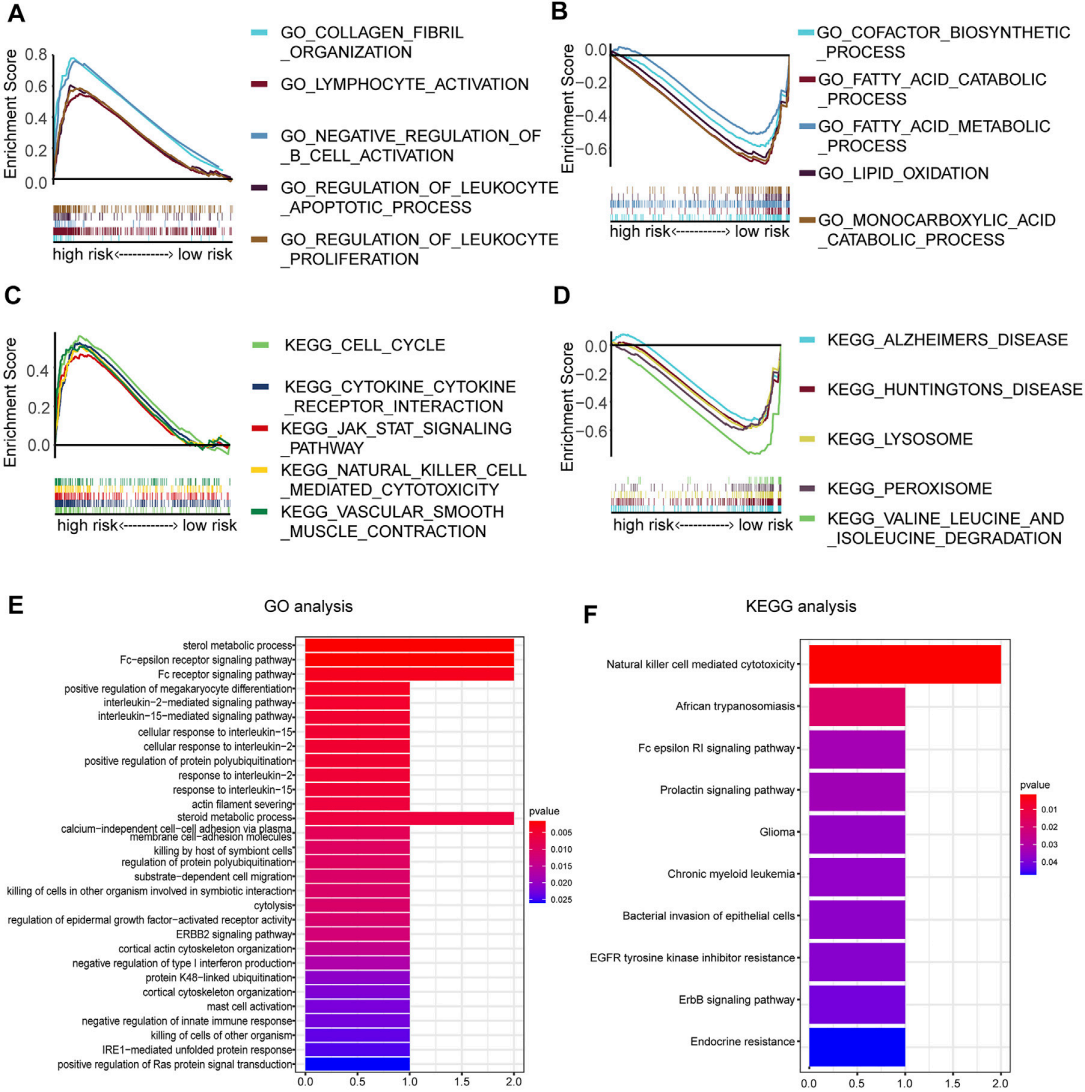
Enrichment analysis of 8 DEMDGs. **(A)** Biological processes enriched in the high-risk groups. **(B)** Biological processes enriched in the low-risk groups. **(C)** Signaling pathways enriched in the high-risk group. **(D)** Signaling pathways enriched in the low-risk group. **(E)** GO enrichment analysis of 8 DEMDGs in biological process. **(F)** KEGG enrichment analysis of 8 DEMDGs.

To further annotate functions enriched in the high- and low-risk groups, GSEA was queried to confirm the signaling pathways in which the genes are enriched. The results are represented in Figures 7A–D. The following biological processes were enriched in the high-risk groups: collagen fibril organization, lymphocyte activation, negative regulation of B-cell activation, regulation of the leukocyte apoptotic process, and regulation of leukocyte proliferation. The following signaling pathways were enriched in the high-risk groups: cytokine–cytokine receptor interaction. To clarify the possible molecular mechanism of eight prognosis-related DEMDGs, we also performed GO and KEGG pathway analysis. Results of the GO analysis were significantly enriched in the sterol metabolic process, Fc-epsilon receptor signaling pathway, Fc receptor signaling pathway, positive regulation of megakaryocyte differentiation, interleukin-2 mediated signaling pathway, etc. (Figure 7E). Results of KEGG analysis were significantly enriched in the natural killer cell-mediated cytotoxicity, African trypanosomiasis, Fc epsilon RI signaling pathway, prolactin signaling pathway, and chronic myeloid leukemia (Figure 7F). These results suggested that RCC patients’ prognosis might be impacted by the above biological functions and signaling pathways.

### The Predictive Accuracy of the Prognostic Model

**FIGURE 8:**
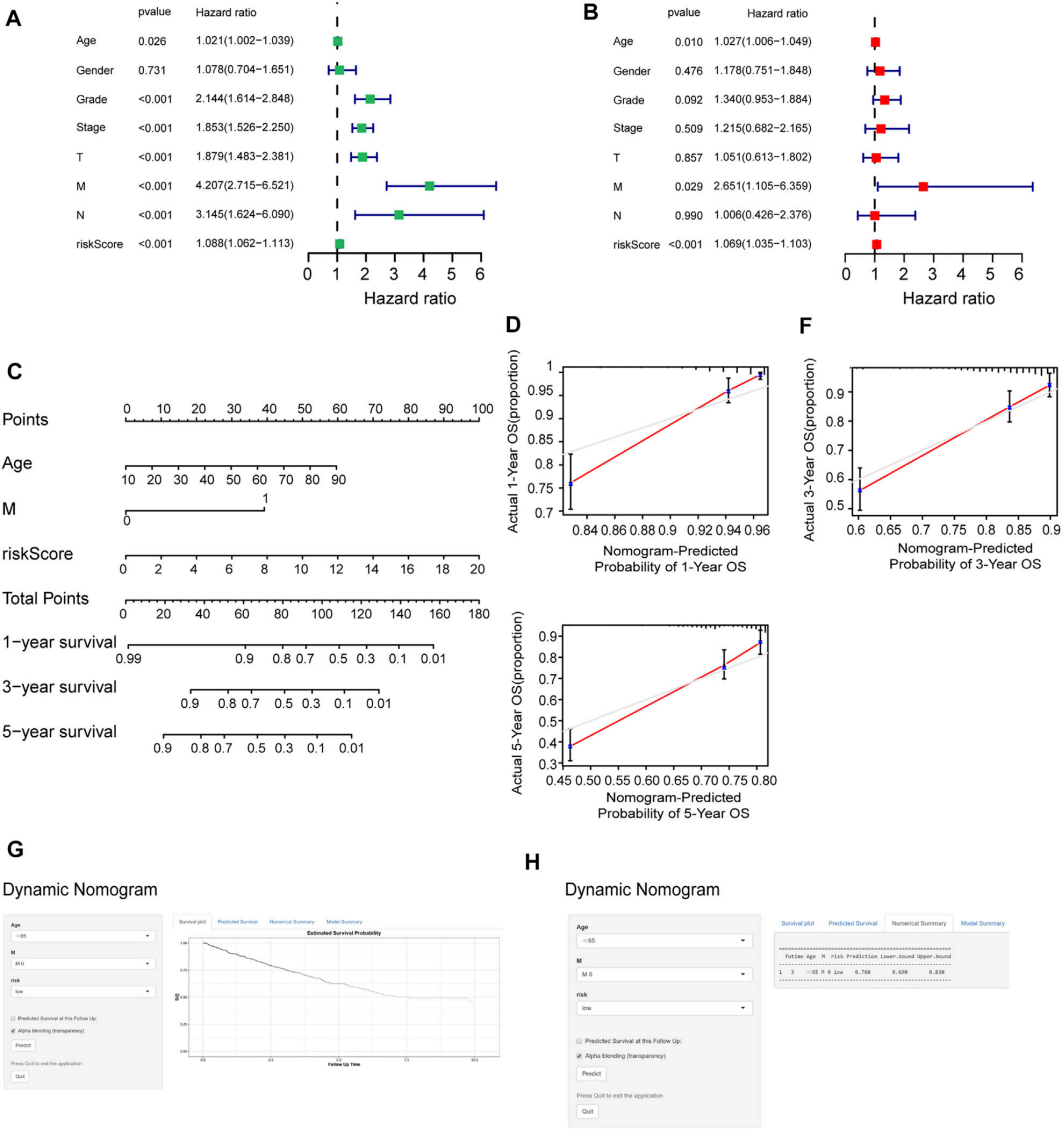
Assessment of the accuracy of the prognostic model. **(A, B)** Univariate **(A)** and multivariate **(B)** Cox regression analyses for the relationship between risk score and clinical features. **(C)** Construction of the nomogram model. **(D–F)** The calibration curves of 1, 3, and 5 years in the nomogram. **(G)** Patients with low-risk, M0 stage, and aged <65 years, according to the web survival rate calculator (95% CI 69–83%). **(H)** 95% confidence interval according to the web survival rate calculator.

To determine whether the risk score could be presented as an independent prognostic factor for RCC patients, we employed univariate and multivariate Cox proportional hazard regression analyses. In the univariate analysis and multivariate analysis, the risk score, age, and M stage showed pronounced effects on the RCC prognosis (*p* < 0.05) (Figures 8A, B). Furthermore, we constructed a nomogram with the significant variables in the multivariate analysis (Figure 8C). Results suggested that the risk score had a significant influence on survival prediction. The 1-, 3-, and 5-year predicted calibration curves also respectively suggested that the model had a good prediction accuracy (Figures 8D–F). We also established a dynamic web-based survival rate calculator (http://127.0.0.1:3496), which could individually predict the survival of patients according to their clinical features and risk score. For example, the 3-year cancer-specific survival rate was approximately 76% (95% CI 42–72%) for patients with low risk, M0 stage, and aged <65 years (Figures 8G, H).

### Prognostic Model Correlated With Tumor-Infiltrating Immune Cells and Drug Susceptibility

**FIGURE 9:**
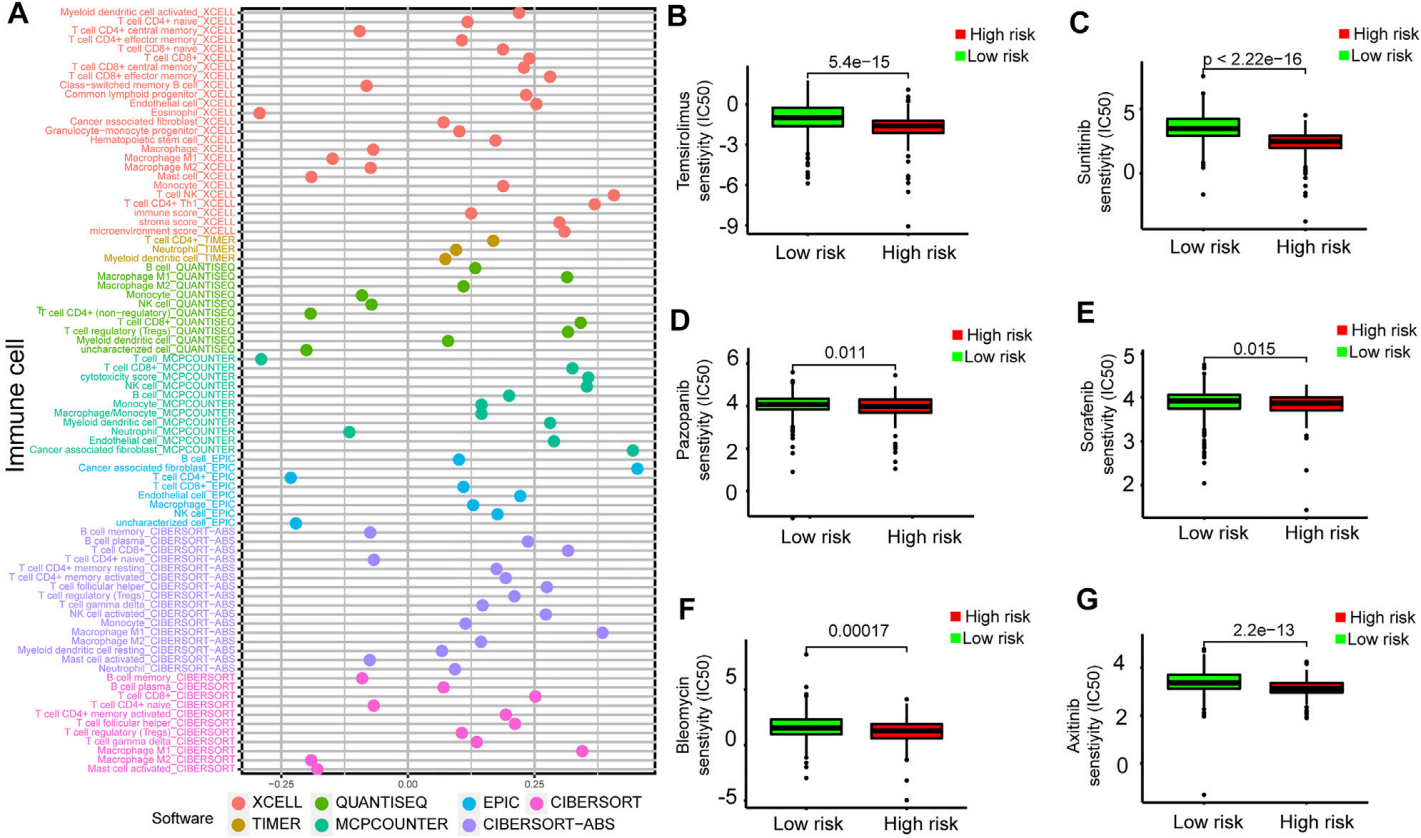
Estimation of tumor-infiltrating cells and drug susceptibility by the prognostic model. **(A)** Spearman correlation analysis between risk score and tumor-infiltrating immune cells. **(B–G)** The model had the potential to predict chemosensitivity because a high risk score was associated with a lower half-maximal inhibitory concentration (IC50) of chemotherapeutics such as temsirolimus (*p* = 5.4e−15), sunitinib (*p* < 2.22e-16), pazopanib (*p* = 0.011), sorafenib (*p* = 0.015), bleomycin (*p* = 0.00017), and axitinib (*p* = 2.2e−13).

To further analyze the relationship between the prognostic model and tumor-infiltrating immune cells, we performed a detailed Spearman correlation analysis, and the result was presented with the lollipop shape (Figure 9A). High-risk groups were more positively correlated with tumor-infiltrating immune cells, including CD8^+^ T cells, macrophage M1, B cells, monocytes, myeloid dendritic cells, regulatory T cells, and myeloid dendritic cells. The detailed results are shown in Supplementary Table S3. We also attempted to identify associations between the prognostic model and the efficacy of six common chemotherapeutic drugs for the treatment of RCC. The high-risk score was associated with the lower half-maximal inhibitory concentration (IC50) of chemotherapeutics such as temsirolimus (*p* = 5.4e−15), sunitinib (*p* < 2.22e-16), pazopanib (*p* = 0.011), sorafenib (*p* = 0.015), bleomycin (*p* = 0.00017), and axitinib (*p* = 2.2e−13) (Figures 9B–G). The aforementioned results showed that this model closely correlated with tumor-infiltrating immune cells and drug susceptibility.

## Discussion

RCC is one of the most common tumors of the urinary system and is occult and insensitive to chemoradiotherapy. Previous studies had described that RCC possesses a high number of genetic alterations and epigenetic alterations (Wang et al., 2017; Chhabra and Nanjundan, 2020). As the major epigenetic modification, DNA methylation studies have become a research hotspot in many cancers, specifically in RCC (Li and Qian, 2019).

In this study, we found that DNA methylation was associated with specific clinical characteristics and hallmark features of RCC. We selected 5,198 DEGs from normal samples and RCC samples. Then, 270 MDGs of RCC were identified by using the MethylMix algorithm. We identified 146 DEMDGs by intersecting 5,198 DEGs and 270 MDGs. The consensus clustering based on 146 DEMDGs could be used to predict the prognosis of RCC, and the clusters were associated with the immune microenvironment of RCC. The RCC patients were successfully divided into two clusters based on the 146 DEMDGs, and patients of different clusters had different clinical features, methylation levels, and gene expression levels. The OS of patients in cluster 2 was significantly longer than those in cluster 1. The immune status, immune score, immune checkpoints, and infiltrating percentage of immune cells in two clusters also showed significant differences. Two clusters had different survival rates for the following possible reasons. 1) Aberrant DNA methylation could contribute to tumor progression due to gene aberrant transcriptional responses (Yang et al., 2019). Aberrant DNA methylation patterns are a feature of tumor development (Xu et al., 2012). 2) Tumor-infiltrating immune cells of RCC could influence the prognosis and progression of tumors (Xing et al., 2020). Additionally, cluster 1 had the higher immune score and immune cell infiltration. Studies were reporting that high immune scores, as well as high infiltration of immune cells, were associated with poor prognosis, which was similar to our results (Deng et al., 2020). We identified 106 DEGs from cluster 1 to perform further analysis. Many results of biological processes were significantly enriched in immunity, including positive regulation of I-kappa B kinase/NF-kappaB signaling and the leukotriene D4 metabolic process. This provided further evidence that the different immune statuses of two clusters may be the possible cause for different survival statuses.

Subsequently, eight DEMDGs that were significantly associated with the prognosis of patients with RCC were also identified. We then constructed an eight DEMDG-related prognostic model to predict the prognosis of stratified patients with RCC. The identified signature was integrated with clinical features to establish the composite prognostic nomogram, which reliably demonstrated accurate prognostic predictions for the patients. Finally, we identified the clinical outcomes, immune infiltration, and drug response features associated with the prognostic signature. Further, qRT-PCR analysis validated the eight DEMDG expressions’ tendency in RCC cell lines. To determine the clinical relevance of eight DEMDGs, HPA clinical specimens were used to analyze the proteins’ expression encoded by these eight genes. The risk score was also calculated based on the gene expression and regression coefficients of each gene. Patients were divided into high-risk groups and low-risk groups based on their risk scores. Patients in the low-risk groups had a longer overall survival than those in the high-risk groups (*p* < 0.001). We used GSEA to confirm the signaling pathways where the genes were enriched in the high- and low-risk groups. The high-risk groups were mainly enriched in immune-related processes. Then, we performed GO analysis and KEGG analysis on the eight DEMDGs. We noted that the BP group of GO analysis was mainly enriched in immune-related processes. These results suggest a close relationship between the prognosis and immune status in RCC. The multivariate Cox regression analysis results indicated that our prognostic model was unaffected by clinical features. We confirmed the prognostic value of the prognostic model built with eight DEMDGs. The risk score of the eight DEMDG-related prognostic model was a stable, independent prognosis factor. Moreover, we established a composite nomogram by integrating the eight DEMDG-related prognostic model with traditional stratifying factors (age and M stage). The dynamic nomogram showed improved prognostic accuracy than the prognostic model. These results indicate that the prognostic model is a powerful tool for predicting the prognosis of patients with RCC.

We further explored the relationship between tumor-infiltrating immune cells and the risk score with seven common acceptable methods of estimating the infiltrating immune cells, including TIMER (Bu F, 2021), CIBERSORT (Bu et al., 2021), xCell (Oshi et al., 2021), quanTIseq (Finotello et al., 2019), MCPcounter (Li et al., 2021), EPIC (Zeng et al., 2021), and CIBERSORT-ABS (Tamminga et al., 2020). The synthetical analysis showed that the risk score was more positively related to tumor-infiltrating immune cells.

Finally, we investigated the relationship between the signature and drug response to promote personalized treatment decisions. To date, immune checkpoint inhibitors have been approved for RCC treatment. However, due to the existence of a highly dynamic, adaptive, and heterogeneous tumor microenvironment and due to the glucose and lipid metabolism in RCC, this cancer may be accompanied by various types of resistance to TKIs and ICIs. Therefore, it is critical to find new biomarkers for appropriate patient selection for immunotherapy. The results showed patients with low risk scores might benefit from these drugs. Our signature may further aid the design of a more reasonable and effective treatment regimen, contributing to precision therapy for individual patients with different risk levels. Here, this study demonstrated a novel prognostic model constructed by eight DEMDGs that could predict prognosis for patients with RCC and might help distinguish those who could benefit from antitumor immunotherapy.

## Conclusion

To summarize, our study found that DNA methylation was associated with specific clinical outcomes and identified 146 DEMDGs in RCC. The eight DEMDGs that were significantly associated with the prognosis of patients with RCC were also identified. We utilized the eight DEMDGs to construct a new OS-related prognostic model for early diagnosis of RCC, and the risk score was significantly correlated with prognosis, immune infiltration, clinical features, and drug sensitivity. The pathway activation underlying the signature was also identified. This study has several strengths. First, we took TCGA dataset and the GTEx dataset together to perform analysis, overcoming the short plank of smaller numbers of normal samples in TCGA dataset. Second, we broke the limitations of previous studies by analyzing all pathological types of RCC instead of the main pathological type. Third, we used the web-based survival rate calculator to validate the predictive accuracy of the prognostic model. However, our study has its limitations as well. First, we used only two clinical characteristics (age and M stage) to establish the composite nomogram. Then, the response of immunotherapy and chemotherapy should be further verified by clinical data in other cohorts. Finally, our findings still need to be demonstrated by experimental methods to further confirm the methylation level of eight mRNAs. We will incorporate this work into future research.

## Data Availability

The original contributions presented in the study are included in the article/Supplementary Material, further inquiries can be directed to the corresponding authors.

## Supplementary Material

Supplementary Figure 1 Heatmap of 106 DEGs in normal samples and RCC samples. Heatmap of 106 DEG expressions in normal samples and RCC samples.

Supplementary Figure 2 Functional enrichment analysis of 106 DEGs. **(A, B)** GO enrichment analysis of 106 DEGs. GO term enrichment in cellular component **(A)** and molecular function **(B)**.

Supplementary Figure 3 Survival analysis on eight DEMDGs based on TCGA dataset. K–M survival analysis of eight DEMDGs in TCGA whole cohort (K–M, Kaplan–Meier).

Supplementary Figure 4 Correlation analysis between the expression levels of eight DEMDGs and the corresponding methylation levels of each gene. **(A–H)** Relation of expression levels and methylation levels of the eight DEMDGs and the corresponding distribution map of the methylation degrees: **(A)** LCP2, **(B)** PPP1R18, **(C)** APOL1, **(D)** FMNL1, **(E)** CLDN7, **(F)** NMI, **(G)** FAXDC2, and **(H)** SHC1.

Supplementary Table 1 Primer sequences involved in this study.

Supplementary Table 2 270 MDGs in RCC samples and normal samples.

Supplementary Table 3 Correlation between the prognostic model and tumor-infiltrating immune cells.

## Abbreviations

CDF, cumulative distribution function; DEGs, differentially expressed genes; DEMDGs, differentially expressed DNA methylation-driven genes; FC, fold change; FDR, false discovery rate; GO, gene ontology; GSEA, gene set enrichment analysis; GTEx, genotype-tissue expression; HRs, hazard ratios; IC50, half-maximal inhibitory concentration; KEGG, Kyoto encyclopedia of genes and genomes; LASSO, least absolute shrinkage and selection operator; MDGs, DNA methylation-driven genes; OS, overall survival; RCC, renal cell carcinoma; TCGA, The Cancer Genome Atlas.
