# Supplementary figures and images for: Molecular Characterization, Tumor Microenvironment Association, and Drug Susceptibility of DNA Methylation-Driven Genes in Renal Cell Carcinoma
Source: Front Cell Dev Biol. 2022 Mar 21;10:837919. doi: 10.3389/fcell.2022.837919 (PMC8978676; doi:10.3389/fcell.2022.837919)

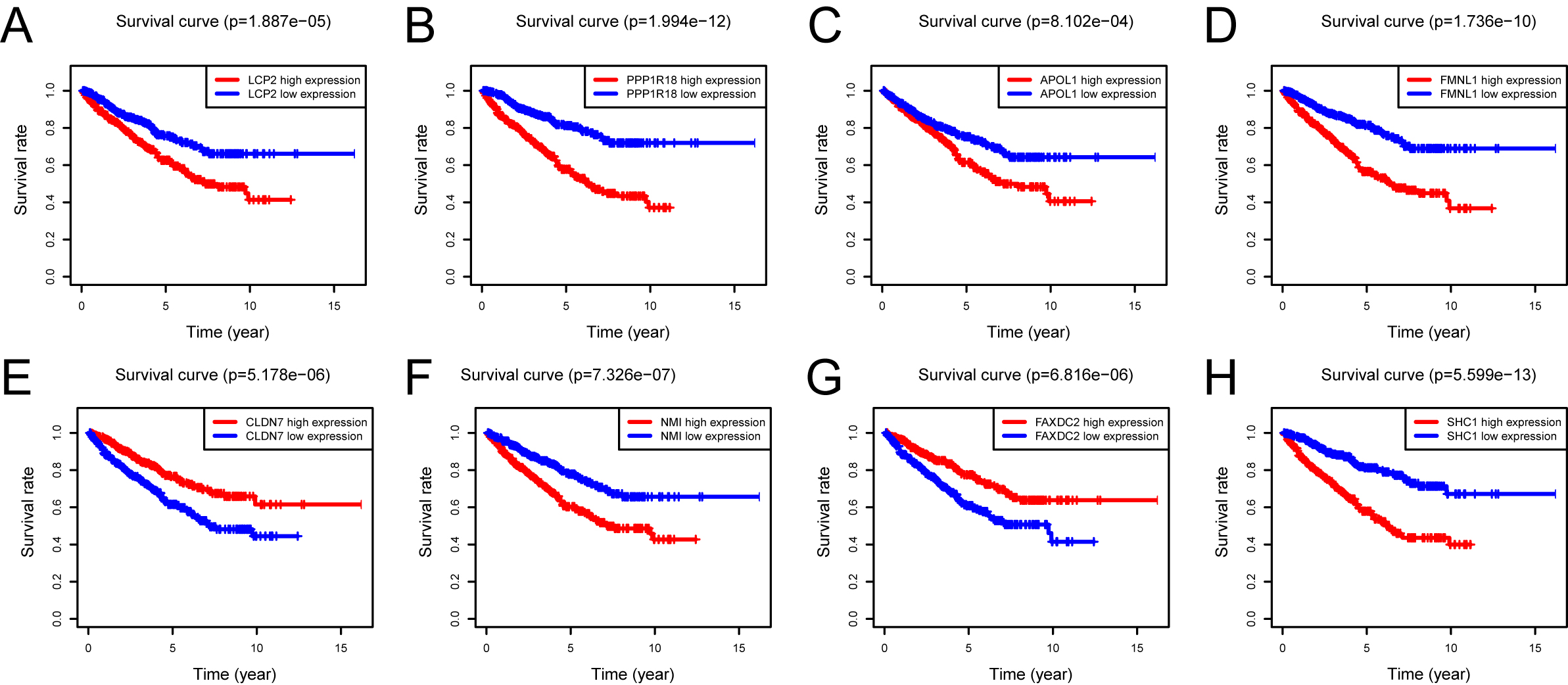

Supplement: Supplementary file 2 [file Image3.JPEG]

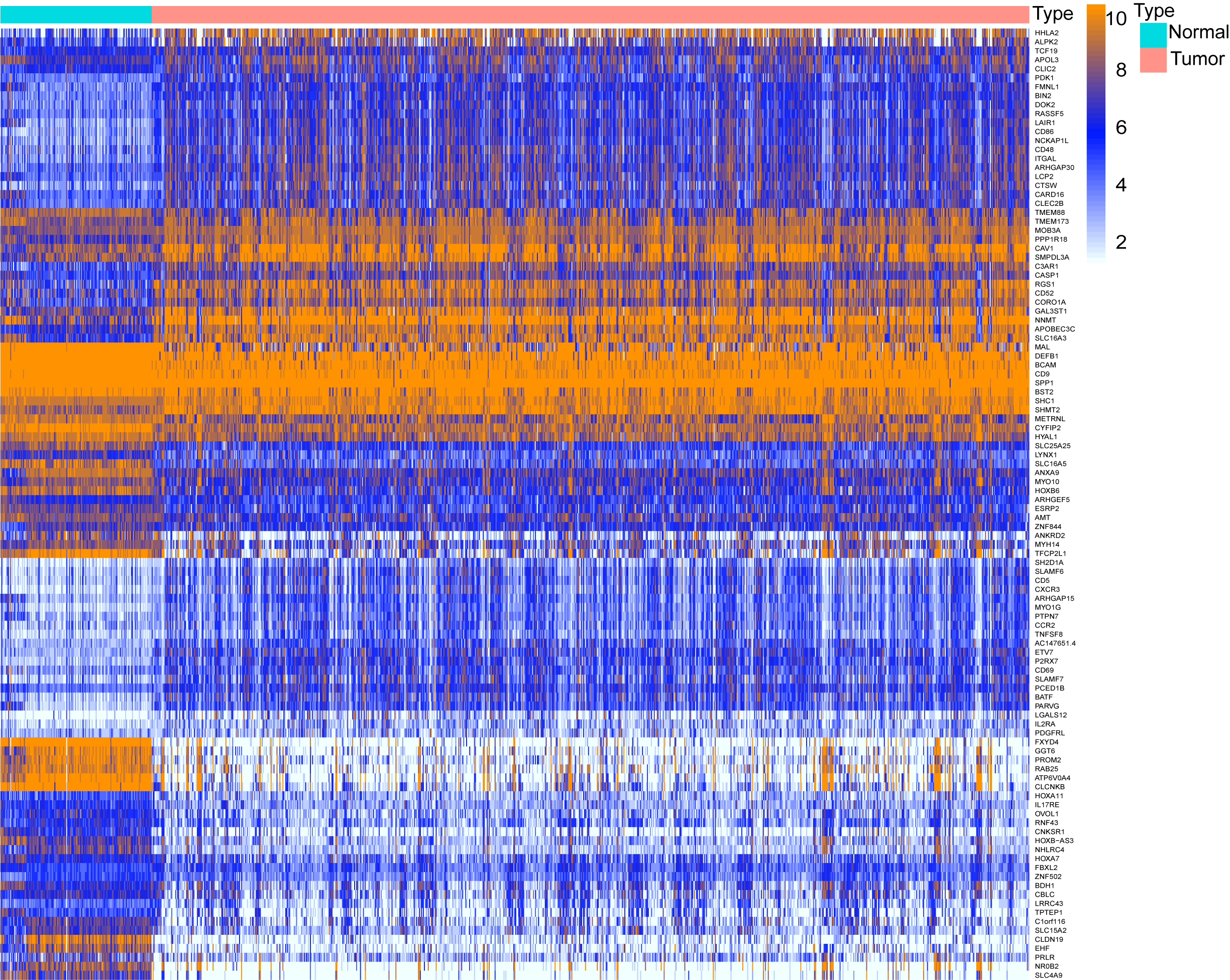

Supplement: Supplementary file 4 [file Image1.JPEG]

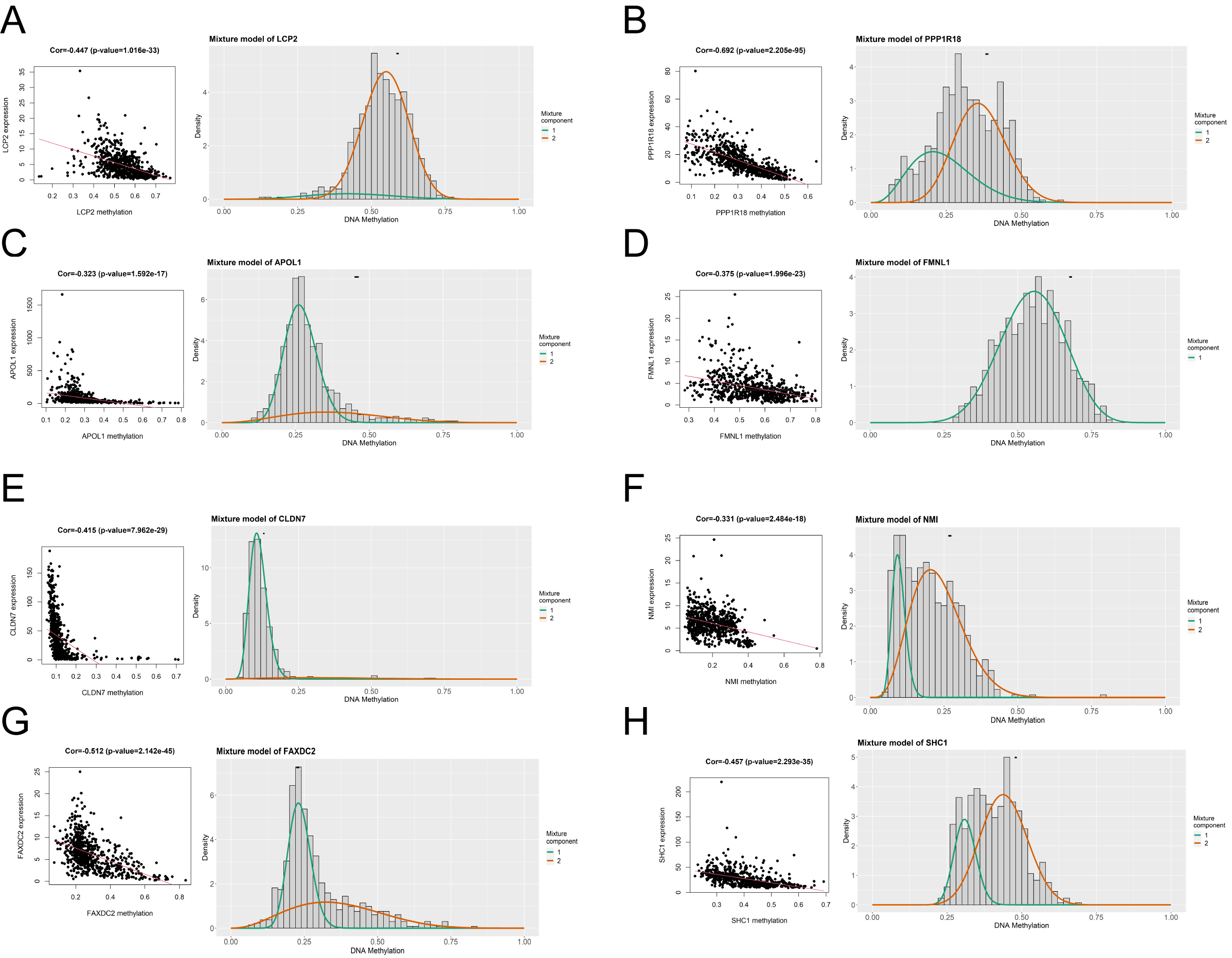

Supplement: Supplementary file 5 [file Image4.JPEG]

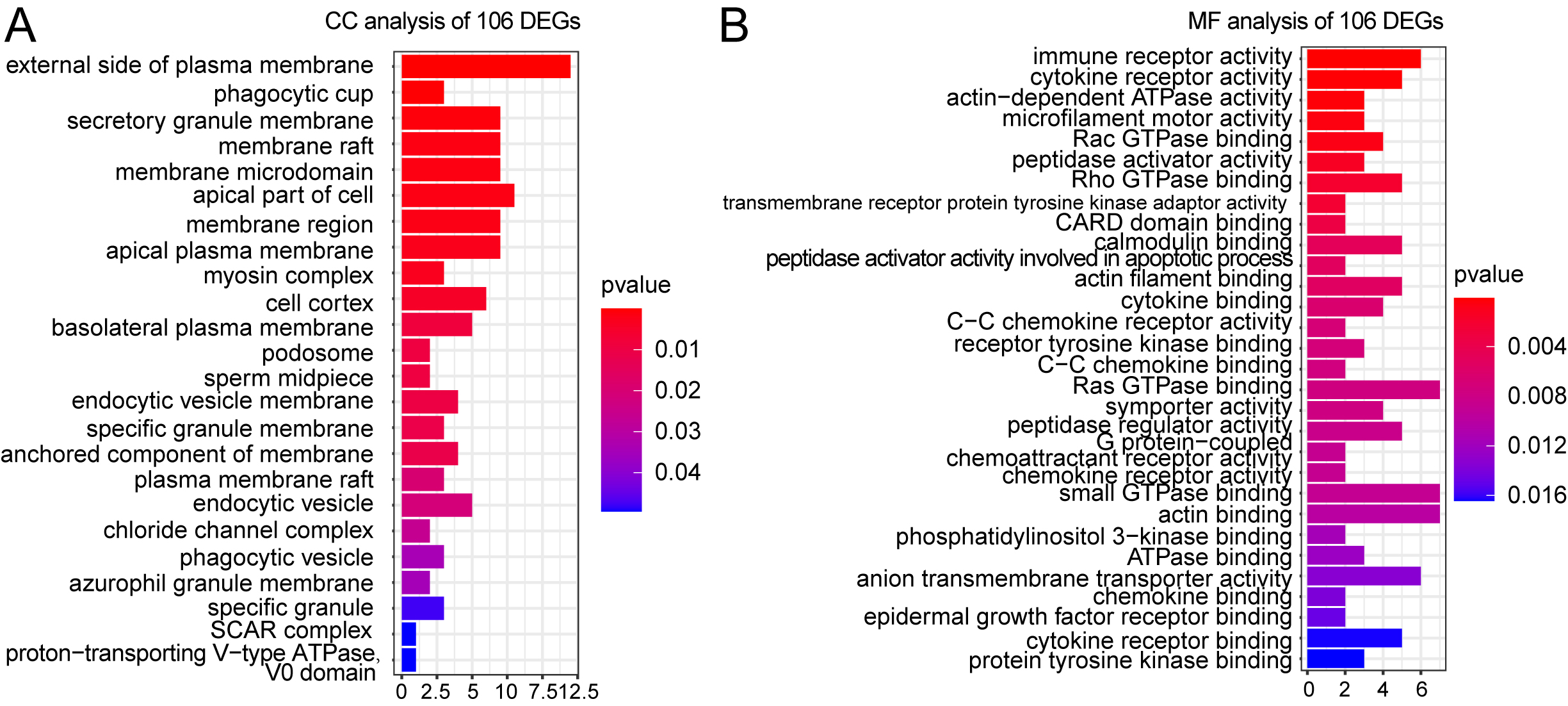

Supplement: Supplementary file 6 [file Image2.JPEG]
